# Obstetrical Practice and Malpractice

**Published:** 1870-12

**Authors:** L. A. Harcourt

**Affiliations:** Chicago, Ill.


ART. III.—*Obstetrical Practice and Malpractice. By* L. A. HARCOURT, M. D., *of Chicago, Ill.*

At seven and one-half o'clock, on the morning of April 1st, 1870, I was called to see Mrs. R., Kinzie St., aged 24 years, in labor with her first child. She had had an abortion about twelve or fourteen months before, and, being attended by a midwife, the placenta had been left in the womb for four days, giving rise to severe inflammation of that organ. It was then expelled by the natural efforts of the womb. In the present case, the lady was attended by the same midwife. Her labor had commenced twenty-four hours before I was called; and the midwife said the pains had become feeble and ceased to do any good about two o'clock the night before. I should mention that the woman expected her confinement three weeks before it took place. Such was the history of the case up to the time I was called in.

I found her in a state of prostration bordering upon collapse, not having slept for 60 hours, nor taken any nourishment since her labor began. Her face was flushed and feverish; pulse weak, rapid and irregular; tongue furred and tremulous. She was harnessed with ropes so as to push with her feet and shoulders, pull with her arms, and bring almost every muscle of the body into action, and thus she had been pulling and trying for twenty-four hours.

A digital examination revealed a partially dilated os, with a soft pulpy, irregular mass presenting. It differed from anything I had ever felt, and puzzled me to know just what it was. At present I did not seek to determine. Had the harness removed, the woman placed in a comfortable position, and prescribed stimulants and anodynes, that she might rally from the exhaustion, and gain a little rest. I then left, promising to return in an hour or two.

Nine p.m. Called again, and found the patient more comfortable. She thought she could rest if left undisturbed. Repeated the anodyne; told her friends not to disturb her, that rest, in her case, was a *sine qua non*, and left to return at daylight next morning, at which time I thought the pains would return. Called at daylight. She had rested pretty well through the night, and the pains had just returned. They were now vigorous, and labor seemed to be progressing. A more careful examination enabled me to determine the presentation which had puzzled me the night previous. The vertex presented in the first position; but the bones of the head had collapsed, allowing the brain substance to protrude, and form the soft, pulpy tumor before mentioned. Told her husband the nature of the case. He could scarcely believe it, as the nurse or the midwife had assured them the night before that the child was living, and the woman herself thought she had felt life. The pains were now efficient; but the tumor, being soft and yielding, would not dilate the os. By careful manipulation, I detached the occipital bone, and used it as an handle to which to make traction. The occipito-frontalis muscle being in a state of partial decomposition, ruptured, and a large portion of the brain substance escaped. It was one putrid mass of corruption, and stunk worse than the concentrated essence of ten thousand skunks. Its removal, however, facilitated the delivery. The cranium was removed piecemeal, its covering still forming a convenient lever by which to make traction. By getting my finger in the axilla, I succeeded in bringing down the left arm and shoulder, then the other, after which the delivery was soon completed. The cord had completely sloughed away, so that when the fœtus was expelled the placenta was left in the womb, without any guide leading to it. After waiting some minutes, believing the pains would not expel the after-birth, and being anxious to finish a disagreeable job, I introduced my hand into the uterus, and without much difficulty removed it, It was also partially decomposed. The fœtus was a monstrosity, at least in size weighing fifteen pounds without the head.

The woman never rallied fairly from the depression. There was no pain, but constant fever, with weak and frequent pulse, from 140 to 160 per minute. Sometimes it could not be counted, and could scarcely be felt. The vital powers seemed to be exhausted by the action of the morbific poison upon the system, which appeared to have been absorbed from the decomposing fœtus, and to have permeated her whole being. There was no power of recuperation left. She lingered two or three days in a semi-comatose condition, and died, from what? I shall leave your readers to determine.

